# Vitamin D Status among First-Generation Immigrants from Different Ethnic Groups and Origins: An Observational Study Using the Canadian Health Measures Survey

**DOI:** 10.3390/nu13082702

**Published:** 2021-08-05

**Authors:** Said Yousef, Douglas Manuel, Ian Colman, Manny Papadimitropoulos, Alomgir Hossain, MoezAlIslam Faris, George A. Wells

**Affiliations:** 1 School of Epidemiology and Public Health, Faculty of Medicine, University of Ottawa, Ottawa, ON K1H 8M5, Canada; icolman@uottawa.ca (I.C.); alhossain@ottawaheart.ca (A.H.); gawells@ottawaheart.ca (G.A.W.); 2 Cardiovascular Research Methods Centre, University of Ottawa Heart Institute, Ottawa, ON K1Y 4W7, Canada; 3 Ottawa Hospital Research Institute, Ottawa, ON K1Y 4E9, Canada; dmanuel@ohri.ca; 4 Institute for Clinical Evaluative Sciences, Ottawa, ON K1Y 4E9, Canada; 5 Department of Family Medicine, Faculty of Medicine, University of Ottawa, Ottawa, ON K1H 8M5, Canada; 6 Eli Lilly Canada Inc., Toronto, ON M5X 1B1, Canada; papadimitropoulos_manny@lilly.com; 7 Faculty of Pharmacy, University of Toronto, Toronto, ON M5S 3M2, Canada; 8 Department of Clinical Nutrition and Dietetics, College of Health Sciences/Research Institute of Medical and Health Sciences (RIMHS), University of Sharjah, Sharjah 27272, United Arab Emirates; mfaris@sharjah.ac.ae or

**Keywords:** vitamin D, serum 25-hydroxyvitamin D, first-generation immigrants, ethnicity, melanin, dietary intake

## Abstract

One in five Canadians are first-generation immigrants. Evidence suggests the baseline risk for vitamin D (vitD) deficiency is increased among immigrants who move from equatorial to northern countries. We investigated the prevalence and determinants of vitD deficiency/insufficiency among first-generation immigrants compared with native-born Canadians and identified explanatory covariables. We used a cross-sectional design with data from the national Canadian Health Measures Survey (Cycles 3 and 4) (11,579 participants aged 3–79 years). We assessed serum 25-hydroxyvitamin D (S-25(OH)D) levels, sociodemographic and environmental factors, immigration status, length of time in Canada, vitD-rich food intake, ethnicity, and place of birth. Immigrants had lower mean S-25(OH)D than non-immigrants (51.23 vs. 62.72 nmol/L, *p* < 0.001). Those with younger age at the time of immigration (<18 years) had a high risk for low vitD, and S-25(OH)D levels increased with the length of time they had lived in Canada. The highest deficiency levels were in immigrants born in Morocco, India, and Lebanon compared with native-born Canadians. Ethnicity was the factor most strongly associated with S-25(OH)D. Compared with the white ethnic grouping, the Japanese had the highest level of vitD deficiency, followed by Arabs and Southeast Asians. Ethnic variations, dietary intake, and lifestyle factors are the main predictors of/explanatory factors for vitD status among Canadian immigrants.

## 1. Introduction

Vitamin D (vitD) plays a crucial role in physiological functions, including skeletal and non-skeletal health [1]. Vit D has two main metabolites, namely 25-hydroxyvitamin D (25-OH) and 1, 25 dihydroxy vitamin D. The dietary sources (vitamin D2 or ergocalciferol) and the animal-based foods (vitamin D3 or cholecalciferol) are the two main forms of vitamin D found in the human body [2,3,4]. The primary source of vitD in the human body, however, is through skin exposure to sunlight (cutaneous synthesis of vitD3) [2,5]. Vitamin D2 and D3 are considered to have equal biological value. The total serum 25-hydroxyvitamin D (S-25(OH)D) concentration is the sum of the 25(OH) D2 and 25(OH) D3 concentrations [6]. The concentration of S-25(OH)D represents the combined contributions of the cutaneous synthesis and dietary intake of vitD and is considered the top clinical marker for overall S-25(OH)D level [7,8,9]. The S-25(OH)D is expressed in nanomoles per liter (nmol/L) and has a stable half-life (up to three weeks) in the human body [7].

The mean of S-25(OH)D (nmol/L) and/or the ranges for the thresholds (a deficiency, <25–30 nmol/L; insufficiency, 25–49 nmol/L; sufficiency, ≥50–<75 nmol/L) were commonly reported and used in studies to describe the status of vitD [8]. The insufficient status (<50 nmol/L) is more frequently used to describe hypovitaminosis D [10]. However, the optimal S-25(OH)D concentration for skeletal health is controversial. The Institute of Medicine (IOM) recommends maintaining S-25(OH)D concentration levels above 50 nmol/L, whereas other experts favor the concentration between 50 to 100 nmol/L [11,12,13].

Multiple factors affect the body’s ability to synthesize vitD, including dietary intake, coexisting disease conditions (especially liver and kidney diseases), and sun-related vitD production (e.g., sun exposure) [8,9,14,15]. Other implicated factors include sociodemographic factors (e.g., socioeconomic status, age, and sex), geographical and environmental factors (e.g., season and latitude), cultural and religious aspects (e.g., clothing and prolonged breastfeeding time), and health and genetic factors (e.g., melanin levels and obesity) [8,10,16]. A global review of evidence from six regions (Europe, North America, Latin America, Asia, the Middle East/Africa, and Oceania) showed the leading risk factors for vitD deficiency were older age, female sex, higher latitude, winter season, darker skin pigmentation, less sunlight exposure, poor dietary habits, and absence of vitD fortification [10].

Hypovitaminosis D is a worldwide public health problem to which immigrants are particularly vulnerable, with a high baseline risk among populations with darker skin who migrate from equatorial regions to northern latitudes [17,18]. Migration is considered a significant risk factor for low S-25(OH)D levels attributable to lifestyle and environmental changes, including dietary intake, physical activity, sun exposure, clothing, and a move from low to high latitude countries [8,17,18]. Immigrants in Canada and other Western countries have a high prevalence of vitD deficiency and insufficiency compared with Western people, with a deficiency prevalence of 19.3–80% among different ethnic minorities [8,15,19]. A meta-analysis of 36 studies reported variation in vitD deficiency among dark-skinned immigrants was attributable to the length of residence in the host country, age at immigration, nutritional barriers, and geographic origins and ethnicities of the studied populations [18]. Moreover, the estimated pooled prevalence of vitD deficiency was 77% (95% confidence interval [CI], 70–83%) after adjustment for latitude [18].

The immigrant population in Canada has increased in recent decades. One in five Canadians is foreign-born, with these people originating from over 200 ethnic groups or origin countries [20]. Research evidence suggests the presence of significant differences in sociocultural contexts and various outcomes between first-generation and second or third generation immigrants [21,22]. Moreover, researchers recommended studying and comparing ethnic groups from the same generation [21,22,23,24].

There is a paucity of information about the status of vitD among Canadian immigrants from different ethnic groups and origins. Therefore, we intend to study first-generation immigrants as identified in the Canadian Health Measures Survey (CHMS) data (foreign-born) to investigate vitD status among immigrants compared with the non-immigrant (native-born) population. Most of the previous studies on immigrants’ vitamin D involved aggregated generations of immigrants (first, second, or more generations), resulting in high heterogeneity in the study population. Moreover, they focused on specific groups from relatively few ethnicities or few countries of origin. However, this study is the first to report vitD status among large sample of first-generation immigrants from 13 major ethnic groups and who were migrated from 153 countries of origin compared with white ethnic group and native-born Canadians.

The study aimed to estimate the prevalence and the leading determinants of vitD deficiency/insufficiency among immigrants from different ethnicities and regions/countries of birth compared with white and native-born Canadians. Moreover, to understand the effects of the environment, lifestyle, and acculturation on immigrants’ vitD, we aimed to investigate vitD status to the length of time after immigration. We hypothesized that significant differences exist in vitD status between immigrant and non-immigrant Canadians, with immigrants having lower vitD status.

## 2. Materials and Methods

The Strengthening the Reporting of Observational Studies in Epidemiology (STROBE) statement was adopted in planning, implementing, and reporting this study [25].

### 2.1. Study Design and Participants

The CHMS is a cross-sectional population-based survey conducted by Statistics Canada in collaboration with Health Canada and the Public Health Agency of Canada [26]. The CHMS provided the first national data on vitD for the Canadian population including immigrants [26]. The CHMS was executed in cycles biannually. Compared to the 2006 Census data and the 2011 National Household Survey, CHMS data has unique information in direct physical health measures, representing the Canadian immigrant population, and addressing the gaps in their existing national information [27].

This study used data for Cycles 3 (2012–2013) and 4 (2014–2015) of CHMS data, which included randomly selected individuals aged 3–79 years [26]. Cycles-3 and -4 were selected as being the most thematic consistent cycles in reporting vitD -rich foods and other vitD determinants than the other cycles of CHMS data. Therefore, we combined Cycles 3 and 4 based on instructions provided by Statistics Canada. By merging the two cycles, we aimed to provide a larger sample size and increase the number of primary sampling units to allow for greater precision in estimating small prevalence and providing more detailed analyses.

For each cycle, Statistics Canada determined the sample size to produce reliable and representative estimates at the national level for sex and age groups. The survey covered approximately 96% of the Canadian population, each cycle collected from sixteen collection sites spread across Canada and stratified into five regions: namely British Columbia, the Prairies (Alberta, Manitoba, and Saskatchewan), Ontario, Quebec, and the Atlantic provinces (Newfoundland and Labrador, Prince Edward Island, Nova Scotia, and New Brunswick). A dwelling stratification stage was applied and followed by a roster list of all persons living in the household, and individuals aged 3 to 79 years were randomly selected [26]. All participants provided written informed consent, and the CHMS survey was approved by the Health Canada Research Ethics Board [26].

The CHMS sample population weight was adjusted for age group and sex across Canada’s five standard geographic regions [27]. It is worth mentioning that except for the total number of subjects included in the analysis, the number of participants in each group and the unweighted results cannot be published due to Statistics Canada’s restrictions policy, thus the results are presented as weighted results. Detailed information about merging the two cycles and the CHMS data are presented in Appendix A and on the Statistics Canada website (http://www.statcan.gc.ca, accessed on 24 February 2021) [28,29].

### 2.2. Measures

The S-25(OH)D was measured using chemiluminescence immunoassay technology (DiaSorin^®^, Ltd., Stillwater, MN, USA). The analytical detection limit for S-25(OH)D was 10–375 nmol/L. Data for S-25(OH)D in the two cycles were extracted and used as the outcome-dependent factors.

We used four cut-offs for S-25(OH)D to identify vitD status as follows: (<30 (nmol/L); (<50 (nmol/L); (<75 (nmol/L); and (≥75 (nmol/L). First, <30 nmol/L is used to identify deficient people. Second, (<50 nmol/L) is a cut-off for the insufficient 25(OH) D, which includes the deficient and insufficient people (it is an accumulating value and not range for the insufficient people). Third, (<75 nmol/L) is a cut-off and accumulating value for deficient, insufficient, and sufficient. Fourth (≥75 (nmol/L), is the “no added value” or the “optimal” as defined by IOM or other experts [8,11,12,13]. The remaining last two cut-offs (<75 nmol/L and ≥75 nmol/L) cover 100% of the total population. We assumed that reporting the prevalence of vitD deficiency, insufficiency, sufficiency, and the no added value or optimal categories using these cut-offs is important for clinicians and readers to see the proportion of participants under each stratified category. Moreover, we used ranges of S-25(OH)D for the above-mentioned cut-offs (<30 nmol/L; 30–49 nmol/L; 50–74 nmol/L and ≥75 nmol/L) for sub-groups of participants.

Independent variables were factors associated with the risk for developing vitD deficiency/insufficiency: immigration status, sex, age, income, education level, body mass index (BMI, kg/m^2^), smoking status, alcohol consumption, age at immigration, length of time in Canada, sun exposure, sunscreen use, season and month of blood sampling, clothing type, physical activity, region and country of birth, ethnicity, skin pigmentation (melanin level), intake of vitD-rich foods, and vitD supplements/medications used.

We created BMI norms for adults aged ≥ 18 years according to Health Canada’s national standards for weight classification [30]. For children aged 3–17 years, body weight classification was based on World Health Organization percentiles [31]. We used the Canadian Physical Activity Guidelines index to classify participants (aged 5–17 years and ≥18 years) as meeting/not meeting physical activity recommendations [32]. The season of blood sampling was categorized by the month of testing: winter (December–February), spring (March–May), summer (June–August), and fall (September–November). Region of birth was classified using five major regions based on the number of participants: Canada and North America; South/Central America and the Caribbean; Europe; Africa; and Asia. We used Statistics Canada’s geographic classifications to identify the country of birth (153 countries; Appendix A), and selected the top 20 countries based on the number of participants (≥30 participants from each) to include in the analysis. These countries were Canada, China, USA, France, Jamaica, UK, Algeria, Mexico, Pakistan, Netherlands, India, the Philippines, Romania, Hong Kong, Germany, Colombia, Morocco, Italy, Iran, and Lebanon. All other countries were grouped into one category (Others). The 153 countries (excluding Canada) were also combined in another category to represent all immigrants. Statistics Canada identifies ethnicity as white and non-white. The non-white group included different major ethnic groups; namely Aboriginal, South Asian, Chinese, Black, Filipino, Latin American, Arab, Southeast Asian, West Asian, Korean, Japanese, multiple ethnicities, and others. As reported by Statistics Canada, melanin levels were used as an indicator for skin pigmentation, with higher ranks indicating darker skin.

The steps used to combine the two cycles and to calculate the variables of interest are described in Table A1, Appendix A.

### 2.3. Statistical Analyses

We summarized the data using numerical and graphical descriptive statistics. All statistical comparisons used the mean (standard error, SE) for continuous variables and proportions with 95% CIs for categorical variables. Data were stratified for analysis based on the variables of interest. To account for the unequal probability of selection and represent an accurate estimate of the Canadian population, we used the survey command, recommended sample weight, and degrees of freedom in the analyses. All results were weighted values. We used continuous values for S-25(OH)D in the linear regression models. A univariate analysis was used to identify independent covariates. Multivariable analyses were performed to clarify the relationship between S-25(OH)D and immigration status and highlight the factors most strongly associated with lower concentration levels of S-25(OH)D among immigrants.

We used the backward elimination method in a linear regression model adjusted for several independent covariates; namely age, sex, household income, education, BMI, season, sun exposure, sunscreen, country of birth, melanin levels, ethnicity, vitD medication and supplements, and food consumption variables. In our analysis, we applied several evaluation models. In which we determined, by excluding variables with a high percentage of missing data (e.g., margarine consumption) and variables that are influenced by S-25(OH)D levels (e.g., serum calcium), improvements in the model’s estimation which better represents the study population compared to other models. Statistical significance was set at *p* ≤ 0.05. Analyses were performed using SPSS version 26.0 (IBM Corp., Armonk, NY, USA) and Stata version 16.0 (StataCorp, College Station, TX, USA).

## 3. Results

### 3.1. Study Description and Participants Characteristics

The analyses were based on the total number of participants in both cycles. The combined response rate at the Canadian level (response rate for all study components like the household visit, blood samples, and activity monitor) for Cycle-3 was 51.7%, and Cycle-4 was 53.7% [33,34]. Demographic characteristics for the two cycles are presented in Table A2, Appendix B.

There were 11,579 participants (5785 from Cycle-3 and 5794 from Cycle-4) aged 3–79 years, with a mean (standard error [SE]) age of 39.23 (0.085) years. S-25(OH)D levels were available for 11,009 participants and were normally distributed with an overall weighted mean of 60.28 nmol/L. Nearly 10.30% of Canadians were vitD deficient, 63.64% had insufficient vitD, 76.1% had sufficient vitD, and 23.90% had optimal vitD status.

Table 1 presents the main characteristics of immigrant and non-immigrant Canadians. Immigrants represented 21.9% of the Canadian population (about 53% female), and the majority were of non-white ethnicity. Compared with the non-immigrant population, they were older (overrepresented in the group aged ≥ 18 years) and had lower household income, smoking, alcohol consumption, BMI (obesity), sun exposure during peak time, and sunscreen use. However, immigrants were more likely to have higher education levels, wear concealing clothing, and have traveled to sunny/warm climates in the two months before blood sampling than non-immigrants.

The weighted mean melanin level (index values) was higher among immigrants than non-immigrants (17.08 vs. 16.29, *p* = 0.004). Immigrants also had lower household income, and serum calcium and phosphorus levels than non-immigrants. Despite the high statistical difference of calcium (*p* = 0.004) and phosphorus (*p* = 0.006), the biological significance of the mean differences between immigrants and non-immigrants for calcium (2.40 vs. 2.42, respectively) and phosphorus (1.32 vs. 1.36, respectively) were negligible (Table 1). Non-immigrants reported more frequent consumption of vitD-rich foods (red/processed meats, liver, milk, cheese, dairy products, fortified margarine, and fatty fish) (Table A3, Appendix B).

### 3.2. S-25(OH)D Concentration and VitD Status by Sociodemographic, Lifestyle, and Immigration Features

Table 2 shows the weighted mean S-25(OH)D levels and vitD status by sociodemographic and lifestyle factors. Youth aged 12–17 years had the lowest S-25(OH)D levels. There were significant differences in sex, BMI (obesity), vitD supplement/medication use, sunscreen use, sun exposure during peak time (<30 min/day) and traveling to a sunny/warm climate in the two months before blood sampling. Differences were also found in income, smoking, alcohol consumption, physical activity, and clothing type as shown in Table A3 (Appendix B).

Immigrants had lower mean S-25(OH)D levels than non-immigrants (62.72 vs. 51.23 nmol/L, *p* < 0.001). Age at immigration was associated with 25(OH)D levels; younger generations (<18 years) had a higher risk for lower vitD than older people (≥18 years). Moreover, years of residency after immigration was associated with S-25(OH)D levels, especially at 5 and 10 years, indicating that immigrants had higher S-25(OH)D levels the longer they had lived in Canada. The mean S-25(OH)D level was higher among sunscreen users than non-users (62.86 vs. 54.78 nmol/L, *p* < 0.001).

Nearly 64% of participants had insufficient S-25(OH)D levels. VitD deficiency was twice as common among immigrants as non-immigrants. Moreover, compared with non-immigrants, more immigrants had insufficient vitD (52.82% vs. 31.75%) and fewer had optimal vitD (13.89% vs. 26.83%; *p* < 0.001). The more years immigrants had lived in Canada, the greater the proportion with optimal levels at 5 (15.41% vs. 7.65%, *p* = 0.002) and 10 (17.37% vs. 7.95%, *p* = 0.022) years after immigration (Table 2). The S-25(OH)D and vitD status by household income, education, smoking habits, alcohol consumption, physical activity, pregnancy, and clothing type are presented in Table A4, Appendix B.

### 3.3. S-25(OH)D Concentration and VitD Status by Participants’ Ethnicity and Country of Origin

**Figure A1:**
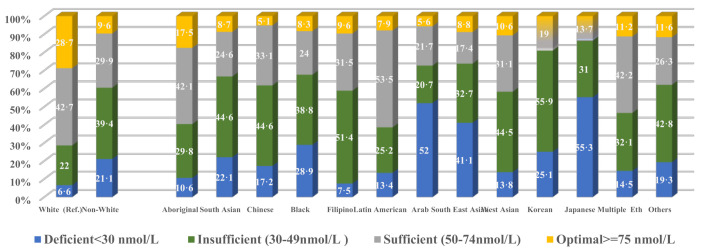
Weighted prevalence of S-25(OH)D status for immigrants from different ethnic groups using Cycles 3 and 4 of Canadian Health Measures Survey data.

Differences were observed in mean S-25(OH)D levels between all ethnic groups compared with the white grouping (mean 64.48 nmol/L), except for the West Asian and Korean groups (Table 3). The lowest mean was reported for the Japanese (33.16 nmol/L; *p* < 0.001), followed by Arabs (37.14 nmol/L; *p* < 0.001), and Southeast Asians (40.92 nmol/L; *p* < 0.001). Compared with the white grouping (6.61%), more than half (55%) of the Japanese ethnic group had vitD deficiency, with similar proportions found for Arabs (52%) and Southeast Asians (41.1%). Moreover, the Japanese had the highest insufficiency level (85%), followed by Koreans (81.0%), and Southeast Asians (73.8%). The ranges for insufficiency (30–49 nmol/L) and sufficiency (50–74 nmol/L) for each ethnic group are also presented in Figure A1, Appendix B.

**Figure A2:**
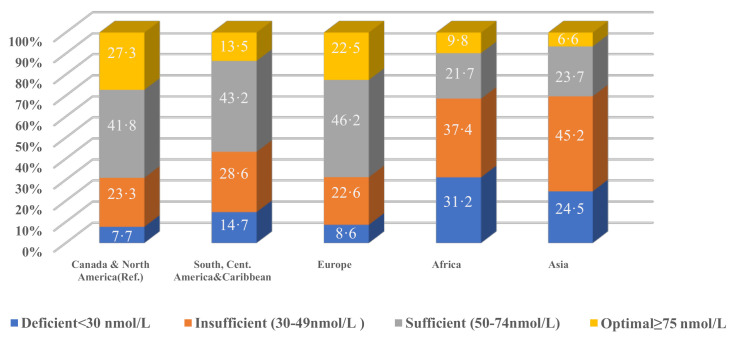
Weighted prevalence of S-25(OH)D status for immigrants by region of birth using Cycles 3 and 4 of Canadian Health Measures Survey data.

Table 4 highlights the marked differences among those born in Africa, Asia, and South/Central America, and Caribbean regions. The highest rates of deficient and insufficient vitD were found among immigrants from Africa (31.2% and 68.6%, respectively) and Asia (24.5% and 69.7%, respectively) compared with those born in Canada and North America (7.7% and 31%, respectively). The ranges for insufficiency (30–49 nmol/L) and sufficiency (50–74 nmol/L) for the above mentioned five regions are presented in Figure A2, Appendix B.

**Figure A3:**
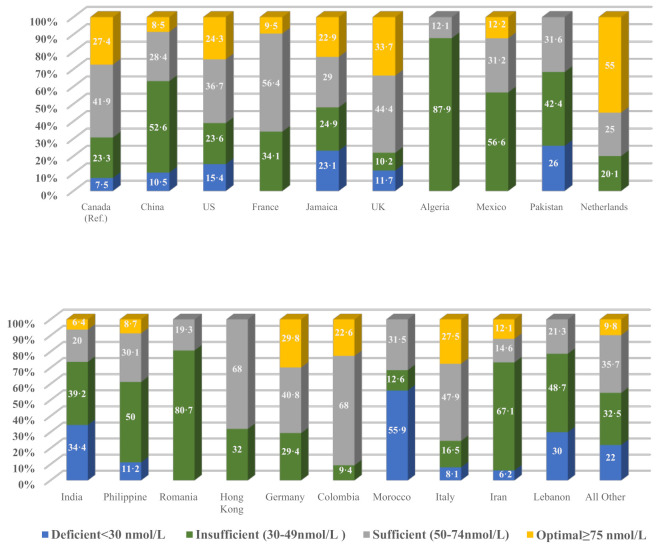
Weighted prevalence of S-25(OH)D status by country of birth using Cycles 3 and 4 of Canadian Health Measures Survey data.

The highest mean S-25(OH)D level was found in those born in the Netherlands, followed by the UK and Germany (72.7, 68.9, and 68.5 nmol/L, respectively). The lowest mean levels of S-25(OH)D were found in those from Algeria (34.7 nmol/L, *p* < 0.001), Lebanon (39.4 nmol/L, *p* = 0.008), and Morocco (39.4 nmol/L, *p* = 0.003). The highest deficient levels were found in those from Morocco (55.9%), India (34.4%), and Lebanon (about 30%), with the highest insufficient levels in those from Algeria (87.9%), Romania (80.7%), and Lebanon (78.7%). The ranges for insufficiency (30–49 nmol/L) and sufficiency (50–74 nmol/L) for each birth country are presented in Figure A3, Appendix B.

### 3.4. S-25(OH)D Concentration and VitD Status by Immigration Status and Season

**Figure 1:**
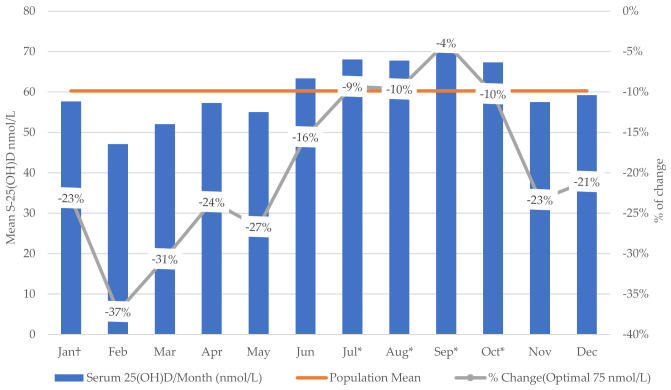
Weighted mean S-25(OH)D (nmol/L)/month of test and percentage of change from the optimal level (75 nmol/L) using Cycles 3 and 4 of Canadian Health Measures Survey data (^†^ reference value * significant at *p* ≤ 0.05).

**Figure 2:**
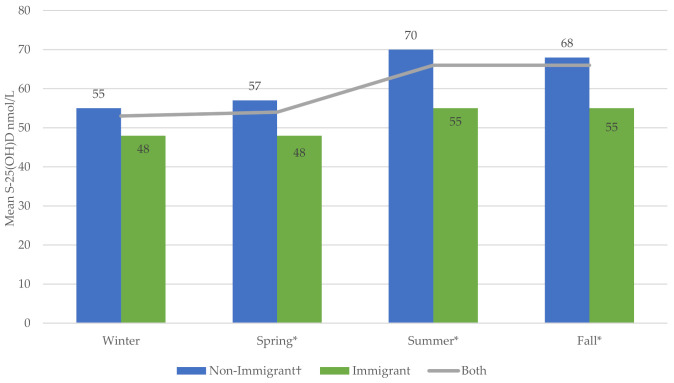
Weighted seasonal variation in mean S-25(OH)D (nmol/L) based on immigration status using Cycles 3 and 4 of Canadian Health Measures Survey data († reference value * significant at *p* ≤ 0.05).

The S-25(OH)D increased substantially during summer and fall compared with winter. Compared with January, the lowest weighted mean was found in February and the highest in September. The highest percentage of change (−37%) from the optimal level (75 nmol/L) was in February (Figure 1) and (Table A5, Appendix B). Non-immigrants had higher S-25(OH)D in all seasons, with significant increments in spring, summer, and fall compared with the winter season (Figure 2) and (Table A6, Appendix B).

### 3.5. Results of Multivariate Analysis

The final multi-linear regression model showed that immigrants had lower S-25(OH)D levels compared with non-immigrants (beta-estimate: −5.28, 95% CI: −7.48, −3.09, *p* < 0.001) after adjusting for all covariates (Table 5). Ethnicity was the strongest predictor of S-25(OH)D in immigrants compared with non-immigrants. Other predictors such as consumption of dairy products (milk, cheese, and yogurt) and traveling to a sunny/warm climate in the two months before blood sampling were also strongly associated with differences in S-25(OH)D. Overall, the model showed a robust association between immigration status and concentrations of S-25(OH)D.

## 4. Discussion

This is the first national Canadian study to report vitD status among immigrants from different ethnic groups and origins compared with non-immigrants and has a global impact. A previous systematic review noted the need to assess vitD status and its determinants (including lifestyle factors) among subgroups living in the same country [35]. Moreover, research has highlighted the importance of comparing the same generation of immigrants rather than an aggregated generation [22], and gathering evidence, and formulating recommendations specific to sub-populations that may differ from the overall immigrant population [16].

The higher overall prevalence of vitD insufficiency among immigrants than non-immigrants in this study was consistent with global evidence suggesting that immigrants in Western countries have lower S-25(OH)D levels than non-immigrant populations [8,10,36,37]. Our multivariate analyses found ethnic background, having traveled to a sunny/warm climate, and low dairy product consumption were strong predictors of low S-25(OH)D levels among immigrants in Canada. Consistent with our findings, associations between S-25(OH)D levels and low income, winter season, low sunlight exposure, conservative dress, BMI (obesity), vitD supplements use, low physical activity, less traveling to a sunny/warm climate, non-white ethnicity, place of birth, and skin pigmentation were reported in previous studies [1,10,18].

While females are usually at higher risk for vitD deficiency [8,36,37,38], females in our study had markedly higher serum S-25(OH)D levels than males. In 2015, Statistics Canada reported that females had more elevated serum of vitD than males, more frequently using supplements than males (41% vs. 28%, respectively), and nearly 85% of vitD supplement users were above the cut-off (<50 nmol/L) compared with non-users (59%) [15]. McCormack et al. 2017, reported that females ≥ 19 years in Canada were using nutritional supplements (including vitD) more than males [39]. However, the higher levels of S-25(OH)D could be partly explained by the finding that females used vitD supplements more than males (66% vs. 34%, *p* = 0.019) (data not shown). In compliance with our finding, studies demonstrated that women who wear concealing clothes had lower S-25(OH)D levels than women dressed according to Western-style. It was well documented that the type of clothing determines the degree of sun exposure and supports/eliminates vitD’s epidermal synthesis [8,10,18,40,41]. In this study, the effect of dress style on S-25(OH)D levels reduced (95% CI: −0.67 15.82; *p* = 0.070) in winter when both immigrants and non-immigrants wear winter clothes that cover all the body, while the difference remained statistically significant for spring, summer, and fall which may be due to different type of clothing during these seasons.

The current study found that immigrants with low household income were more linked with lower S-25(OH)D levels. A global vitD review reported that higher family income in developing countries was found inversely associated with hypovitaminosis D [10]. Other studies confirmed that lower-income immigrants were found with lower levels of S-25(OH)D than higher incomes [42,43]. Consistent with our findings, global and Canadian vitD studies reported that obese adults had significantly lower S-25(OH)D than normal/underweight and overweight [1,8,15,44]. Studies suggested that this may be due to vitD displacement in adipose tissue, which results in lower circulating S-25(OH)D levels in the blood [8].

Dairy products contain essential nutrients including vitD and calcium. Previous studies suggested insufficient consumption of these products was strongly associated with lower S-25(OH)D levels [10,42,45,46]. Furthermore, milk in Canada is fortified with vitD, indicating a possibly magnified contribution of dairy products to serum vitD for both immigrant and non-immigrant consumers. Nonetheless, consumption of fortified milk and dairy products was relatively higher in non-immigrants than immigrants, which may partially explain the difference in S-25(OH)D levels.

Canada regions have different weather conditions; nonetheless, it is expected that days in fall and winter are shorter than in summer and spring, with fewer hours of sunlight overall in Canada. In the present study, immigrants had less sun exposure than non-immigrants and had lower S-25(OH)D levels in winter than in summer. The seasonal variation in S-25(OH)D between immigrants and non-immigrants may be explained by the role of ultraviolet-B (UVB) light exposure in the endogenous synthesis of vitD. Previous studies also reported seasonal variations in vitD status [10,19,47]. Other studies reported the lowest levels of S-25(OH)D in winter [8,48], which was consistent with our findings.

The non-sunscreen users had a higher prevalence of deficiency and insufficiency in vitD status than did the frequent sunscreen users. However, such difference could be partly explained by the fact that sunscreens are used as photoprotector as it minimizes ultraviolet-B (UVB) light exposure, hence lowers endogenous vitD activation, and thus increases the likelihood of developing vitD deficiency [49]. Nonetheless, in practice, sometimes the irregular use of sunscreen or inadequate amounts may not be enough to secure the body from all UVB light. Furthermore, sunscreen users might feel sunburn protected by blocking UVB light and, hence, tend to expose themselves more to the sun than non-users, increasing S-25(OH)D levels. In line with our finding, 25% of studies in a recent review concluded that sunscreen use was associated with higher S-25(OH)D concentration, while 10% of studies reported lower levels and 35% with no association [49,50]).

Our data were obtained from immigrants from more than 150 countries, over 13 major ethnic groups, and therefore present a unique contribution to understanding Canada’s ethnocultural diversity concerning vitD status. Irrespective of the length of time living in Canada, immigrants who were born in Africa and Asia had lower S-25(OH)D levels than those born in North America. The lowest levels by country of birth were among immigrants born in Algeria, Lebanon, and Morocco. In terms of ethnic origins, the lowest S-25(OH)D levels were in Japanese, followed by Arab and Southeast Asian ethnic groups. Our adjusted linear regression model indicated ethnicity was the most important indicator of low S-25(OH)D levels among immigrants compared with non-immigrants. Similarly, previous research reported vitD deficiency was more common in people from specific racial backgrounds. For example, Asian (South Asia, Southeast), African, and Middle Eastern immigrant populations had lower S-25(OH)D than their counterparts in Western countries [8,10,48]. Middle Eastern immigrants (including Lebanese and Iranian people) were more frequently reported to have deficient/insufficient levels than other ethnic groups and non-immigrant populations in Western countries [10]. A systematic review of 112 studies (168,389 participants from 44 countries) reported insufficient levels in one-third of the included studies, with the highest levels among participants living in North America and lower levels among those living in the Middle East and African regions [35].

Dark-skinned individuals have a rich amount of melanin, which acts as a biological shield against UV radiation [8]. In the present study, skin pigmentation was used to rank participants based on melanin index values, where higher degrees of melanin indicated darker skin pigmentation. We found that immigrants had higher melanin levels than non-immigrants. Brook et al. reported the median 25(OH)D concentrations were higher in whites compared with non-whites in Canada [51] and several other studies have documented associations between skin pigmentation, ethnicity, geographical origins, and S-25(OH)D levels [8,18,52]. Research suggests that dark-skinned immigrants are at higher risk for vitD deficiency the longer time they spend in the host country [18]. In contrast, we found immigrants had markedly higher S-25(OH)D levels the longer they had lived in Canada after immigration. However, integration into a new life in Canada may reflect physical and behavioral adaptation to the surrounding environment, including lifestyle, diet, and climate changes. Immigration studies on acculturation and vitD found the length of time since immigration was a crucial indicator of lifestyle acculturation, and higher acculturation levels were associated with significantly higher S-25(OH)D levels [21,53]. However, we found immigrants had less sun exposure and less frequent consumption of vitD-rich foods than non-immigrants, and the length of time since immigration reflected reasonable lifestyle acculturation in Canada. Moreover, genomic studies showed that vitD interacts and influences the human genome through vitD receptor-mediated gene regulation and drives evolution for genomic adaptations in the context of skin color lightening [54]. The biological adaptation process is part of micronutrient gene-regulation that supports vitD3 synthesis in regions with lower UVB radiation [54]. Therefore, epigenetic adaptations may partially explain why immigrants in Canada were prone to vitD deficiency in the short term after immigration, and levels of S-25(OH)D increased over time. Furthermore, our study showed that ethnic differences played a fundamental role in S-25(OH)D levels. Common genetic polymorphisms in the vitD receptor and vitD-binding protein may partly explain differences in vitD status between different ethnicities [55]. Therefore, vitD insufficiency and deficiency among immigrants may be associated with genetic variations combined with dietary and other environmental factors; this issue warrants further investigation.

To our knowledge, this was the first study assessing S-25(OH)D levels among Canadian immigrants’ different ethnic groups and origins compared with the non-immigrant population at the national level. The findings highlighted the need for further research investigating immigrants’ health deterioration in the context of vitD. In addition, building knowledge of the relationship between health deterioration and vitD status along with similar biomarkers has important implications for responding to chronic and infectious diseases, including COVID-19.

Our study should be interpreted with consideration of its strengths and limitations. The CHMS data have many strengths. First, the CHMS provided the first national, comprehensive, and representative data for the Canadian population on vitD. Moreover, it used several clinical and laboratory measures to overcome the limitations of self-reported data [27]. Second, the study design and large sample size provided a nationally representative sample of immigrants in Canada. Third, it captured the ethnocultural diversity of immigrants from more than 150 countries and represented more than 13 major ethnic groups. Fourth, reported melanin level is a reliable indication of skin pigmentation. Fifth, S-25(OH)D levels were collected over the entire year, which enabled adjustment for seasonal variations in UVB light exposure, and offered a more accurate and representative measure of S-25(OH)D at the national level. Finally, the data were validated against other national data such as the Canadian Community Health Survey, Census data, and the 2011 National Household Survey [27]. However, immigrants were identified as landed people without detailed information about the immigration-specific status (e.g., refugees or asylum seekers) [27]. Research suggests refugees have a higher risk for vitD deficiency than other population groups [18,19,56,57]. The lack of data on refugees and asylum seekers limited further subgroup analyses. Another limitation of CHMS data is that the levels of biologically activated form 1,25(OH)2Dwas not measured in the study participants.

## 5. Conclusions

In conclusion, vitD deficiency is more common among Canadian immigrants than non-immigrants. Immigrants originating from African (Algeria and Morocco) and Asian (Lebanon) countries have a particularly low mean of S-25(OH)D. This study highlights ethnicity as a pivotal predictor of immigrants’ lower S-25(OH)D levels; the Japanese, Arab, and Southeast Asian ethnic groups have a high prevalence of vitD deficiency. Short-term prevention strategies in community and clinical settings may be warranted, including vitD supplementation and awareness for people at higher risk. A long-term intervention plan may include developing/updating Canadian immigrant guidelines to incorporate evidence-based improvement strategies for vitD implementation.

## Figures and Tables

**Table 1:** Weighted prevalence of immigrants and non-immigrants by sociodemographic and lifestyle factors using Cycles 3 and 4 of Canadian Health Measures Survey data.

		Non-Immigrants (78.9%), %	Immigrants (21.9%), %	All Participants (100%), %	*p*-Value
Sex	Female	49.37	52.85	50.13	0.142
Age (years)	<5	2.84	0.42	2.31	<0.001
	5–11	9.37	3.38	8.05	
	12–17	8.20	4.57	7.41	
	18–64	69.10	75.41	70.48	
	>64	10.49	16.22	11.75	
Household income (CAD)	<50,000	33.15	43.93	35.51	0.008
	50,000–100,000	37.07	34.65	36.54	
	>100,000	29.79	21.43	27.95	
Education	>Secondary school	47.26	64.63	51.07	<0.001
BMI (kg/m^2^)	Underweight	2.19	2.34	2.22	0.023
	Normal weight	41.40	41.75	41.48	
	Overweight	30.21	37.01	31.70	
	Obese	26.21	18.89	24.60	
Ethnic group	Non-white	11.76	62.67	22.91	<0.001
VitD-supplement and/or analog use	Yes	5.39	4.84	5.27	0.664
Smoking status	Current smoker	22.65	14.75	20.79	0.003
Alcohol	Current drinker	82.90	65.03	78.69	<0.001
Meet the physical activity recommendations	Yes	35.41	30.17	34.21	0.127
Sun exposure (10 am to 4 pm)	≥30 min/day	91.21	78.59	88.46	<0.001
Sunscreen use	Yes	73.94	58.57	70.64	<0.001
Clothing	Typically covered	34.66	52.54	38.51	<0.001
Traveled to sunny/warm climate	Yes	10.56	16.48	11.85	0.018
Weighted means		Mean (SE)	Mean (SE)	(95% CI)	*p*-value
	Age (years)	37.51 (0.26)	45.17 (0.58)	(−9.05, −6.26)	<0.001
	Income (CAD)	91,985 (3831)	76,317 (4004)	(8087, 23,248)	<0.001
	Calcium (mmol/L)	2.42 (0.00)	2.40 (0.01)	(0.01, 0.04)	0.004
	Phosphorus (mmol/L)	1.36 (0.01)	1.32 (0.01)	(0.01, 0.06)	0.006
	Melanin (index values)	16.29 (0.29)	17.08 (0.25)	(−1.29, −0.28)	0.004

BMI, body mass index; CAD, Canadian dollars; SE, standard error; CI, confidence interval. *p*-value: *p* ≤ 0.05.

**Table 2:** Weighted mean S-25(OH)D (nmol/L) and vitamin D status by sociodemographic and lifestyle factors using Cycles 3 and 4 of Canadian Health Measures Survey data.

		S-25(OH)D (nmol/L)			S-25(OH)D Status			
		Mean (SE)	(95% CI)	*p*-Value	<30 (nmol/L (10.3%),%	<50 (nmol/L (63.64%),%	<75 (nmol/L (76.1%),%	≥75 (nmol/L (23.9%),%
Immigration status	Non-immigrant ^†^	62.72 (1.73)	-		7.82	31.75	73.21	26.83
	Immigrant	51.23 (1.41)	(8.37, 14.62)	<0.001	19.01 ***	52.82 ***	86.11	13.89 ***
Age at immigration (years)	<18 ^†^	46.54 (1.63)	-		19.17	64.04	91.37	8.63
	≥18	56.33 (2.34)	(−15.14, −4.44)	0.001	18.80	41.23 **	80.76	19.24 **
5 years after immigration	≤5 ^†^	45.94 (2.22)	-		19.55	62.54	92.35	7.65
	>5	52.77 (1.73)	(−12.58, −1.08)	0.022	18.59	50.04 *	84.59	15.41 *
10 years after immigration	≤10 ^†^	47.03 (1.83)	-		19.55	59.83	92.05	7.95
	>10	53.98 (1.83)	(−11.79, −2.12)	0.007	18.59	48.26	82.63	17.37 **
Sex	Male ^†^	57.92 (1.67)	-		11.13	39.65	79.62	20.38
	Female	62.53 (1.82)	(−6.30, −2.93)	<0.001	9.45	33.04 ***	72.58	27.42 ***
Age group (years)	<5	69.94 (1.64)	(−13.94, −9.01)	<0.001	1.99	15.70	67.1	32.9
	5–11	62.49 (2.16)	(−5.91, −2.29)	<0.001	4.04	27.41	77.9	22.1
	12–17	55.76 (1.93)	(0.44, 4.81)	0.021	10.49	40.38	84.36	15.64
	18–64 ^†^	58.45 (1.81)	-		12.1	39.79	78.24	21.76
	>64	71.07 (1.20)	(−15.63, −9.55)	<0.001	4.59	22.39 ***	58.69	41.31 ***
BMI (kg/m^2^)	Underweight	62.19 (4.05)	(−5.82, 7.51)	0.795	12.65	33.0	71.07	28.93
	Normal weight ^†^	63.03 (2.04)	-		9.22	31.79	73.07	26.93
	Overweight	61.02 (1.68)	(−0.56, 4.58)	0.119	7.91	35.32	75.24	24.76
	Obese	54.48 (1.74)	(5.50, 11.60)	<0.001	15.25 ***	45.15 ***	82.36	17.64 **
VitD-supplement and/or analog use	No ^†^	56.97 (1.74)	-		40.52		80.56	19.44
	Yes	83.46 (5.25)	(−35.92, −17.06)	<0.001	9.80 ***		43.16	56.84 ***
Sun exposure (10 am to 4 pm)	<30 min/day ^†^	55.92 (2.20)	-		15.58	48.72	76.51	23.49
	≥30 min/day	60.82 (1.69)	(−7.81, −1.99)	0.002	9.60 **	34.65 ***	76.04	23.96
Sunscreen use	Never ^†^	54.78 (1.85)	-		15.04	48.05	80.92	19.08
	Always, occasionally	62.86 (1.74)	(−10.16, −5.99)	<0.001	7.87 ***	30.23 ***	73.99	26.01 ***
Traveled to sunny/warm climate	No ^†^	59.33 (1.83)	-		10.81	37.99	77.59	22.41
	Yes	67.03 (2.10)	(−11.85, −3.54)	0.001	6.49	23.71 ***	65.08	34.92 ***

Note: Following the Statistics Canada guideline, some cells merged due to the low number of participants. ^†^ Reference value; SE, standard error; CI, confidence interval; BMI, body mass index. *p*-values: * *p* ≤ 0.05; ** *p* ≤ 0.01; *** *p* ≤ 0.001.

**Table 3:** Weighted mean S-25(OH)D (nmol/L) and vitamin D status by ethnicity using Cycles 3 and 4 of Canadian Health Measures Survey data.

		S-25(OH)D (nmol/L)			S-25(OH)D Status			
		Mean (SE)	(95% CI)	*p*-Value	<30 (nmol/L (10.3%),%	<50 (nmol/L (63.64%),%	<75 (nmol/L (76.1%),%	≥75 (nmol/L (23.9%),%
White ^†^		64.48 (1.73)	-		6.61	29.10	71.25	28.75
Non-white		47.67 (1.31)	(13.85, 19.77)	<0.001	21.09 ***	60.49 ***	90.39	9.61 ***
	Aboriginal	55.55 (1.77)	(4.36, 13.50)	0.001	10.63	40.35 **	82.51	17.49 *
	South Asian	45.56 (2.48)	(13.83, 24.02)	<0.001	22.11 ***	66.73 ***	91.28	8.72 ***
	Chinese	46.03 (1.56)	(14.17, 22.74)	<0.001	17.18 ***	61.84 ***	94.89	5.11 ***
	Black	43.82 (2.96)	(15.07, 26.26)	<0.001	28.87 ***	67.72 ***	91.73	8.27 ***
	Filipino	49.39 (3.93)	(5.90, 24.29)	0.003	7.51	58.85 *	90.41	9.59 ***
	Latin American	52.64 (2.87)	(5.20, 18.50)	0.001	13.43	38.66	92.13	7.87 *
	Arab	37.14 (4.62)	(18.15, 36.54)	<0.001	51.98 ***	72.17 ***	94.40	5.60 ***
	Southeast Asian	40.92 (4.43)	(13.88, 33.24)	<0.001	41.08 ***	73.84 ***	91.19	8.81 **
	West Asian	53.74 (6.64)	(−3.29, 24.78)	0.127	13.83	58.27 **	83.35	16.65
	Korean	44.26 (15.06)	(−10.07, 50.51)	0.180	25.12 *	81.04 ***	18.96	
	Japanese	33.16 (8.59)	(14.12, 48.52)	0.001	55.30 ***	86.27 ***	13.83	
	Multiple ethnicities	53.04 (2.44)	(6.45, 16.44)	<0.001	14.48 **	46.58 ***	88.83	11.17 ***
	Other ethnicities	50.02 (5.17)	(3.33, 25.61)	0.013	62.13 ***	88.36	11.64	62.13 ***

Note: following the Statistics Canada guideline, some cells merged due to the low number of participants. ^†^ Reference value; SE, standard error; CI, confidence interval. *p*-values: * *p* ≤ 0.05; ** *p* ≤ 0.01; *** *p* ≤ 0.001.

**Table 4:** Weighted mean S-25(OH)D (nmol/L) and vitamin D status by geographical region and country of birth using Cycles 3 and 4 of Canadian Health Measures Survey data.

		S-25(OH)D (nmol/L)			S-25(OH)D Status			
		Mean (SE)	(95% CI)	*p*-Value	<30 (nmol/L (10.3%),%	<50 (nmol/L (63.64%),%	<75 (nmol/L (76.1%),%	≥75 (nmol/L (23.9%),%
Region of birth	Canada and North America ^†^	63.10 (1.74)	-	-	7.66	30.95	72.74	27.26
	South/Central America, and the Caribbean	53.20 (3.13)	(2.47, 17.33)	0.011	14.65	43.29	86.51	13.49
	Europe	62.61 (1.83)	(−2.98, 3.95)	0.775	8.62	31.24	77.46	22.54
	Africa	42.74 (3.50)	(13.08, 27.64)	<0.001	31.16 ***	68.55 ***	90.21	9.79 **
	Asia	43.67 (1.67)	(15.51, 23.34)	<0.001	24.50 ***	69.67 ***	93.41	6.59 ***
Country of birth	Canada ^†^	63.23 (1.74)	-		7.45	30.73	72.64	27.36
	China	48.62 (2.08)	(10.21, 19.01)	<0.001	10.53	63.14 ***	91.50	8.50 ***
	USA	58.67 (4.07)	(−4.36, 13.47)	0.301	15.44 *	39.01	75.69	24.31
	France	56.98 (5.19)	(−4.17, 16.67)	0.226	34.10		90.52	9.48
	Jamaica	51.72 (7.47)	(−5.03, 28.05)	0.163	23.13 *	48.04	77.07	22.93
	UK	68.88 (3.16)	(−12.11, 0.81)	0.083	11.73	21.95	66.34	33.66
	Algeria	34.73 (6.35)	(15.49, 41.50)	<0.001	87.90 ***		12.10	
	Mexico	45.67 (11.69)	(−5.28, 40.40)	0.125	56.61		87.81	12.19
	Pakistan	41.60 (7.35)	(4.35, 38.90)	0.017	25.96 *	68.38	31.60	
	Netherlands	72.69 (5.75)	(−20.12, 1.20)	0.079	20.06		45.01	54.99 ***
	India	42.40 (4.69)	(11.64, 30.02)	<0.001	34.42 ***	73.64	93.64	6.36 ***
	Philippine	48.14 (3.55)	(7.25, 22.92)	0.001	11.20	61.20 *	91.32	8.68 **
	Romania	42.95 (5.25)	(9.26, 31.29)	0.001	80.72 ***		19.30	
	Hong Kong	53.90 (7.90)	(−8.08, 26.74)	0.278	32.02		68.00	
	Germany	68.53 (6.84)	(−19.37, 8.77)	0.443	29.44		70.19	29.81
	Colombia	60.53 (9.88)	(−19.08, 24.48)	0.800	9.39		77.36	22.64
	Morocco	39.39 (7.25)	(8.77, 38.90)	0.003	55.90 ***	68.53 ***	31.50	
	Italy	63.12 (5.43)	(−10.52, 10.73)	0.984	8.07	24.55	72.49	27.51
	Iran	49.99 (6.87)	(−1.77, 28.23)	0.081	6.18	73.32 **	87.94	12.06
	Lebanon	39.38 (8.18)	(6.89, 40.80)	0.008	29.97 **	78.70 **	21.30	
	Others	49.28 (1.57)	(10.58, 17.31)	<0.001	21.97 ***	54.45 ***	90.19	9.8%
	All (153 Countries)	51.09 (1.40)	(9.01, 15.27)	<0.001	18.91 ***	53.42 ***	86.66	13.34 ***

Note: following the Statistics Canada guideline, some cells merged due to the low number of participants. ^†^ Reference value; SE, standard error; CI, confidence interval. *p*-values: * *p* ≤ 0.05; ** *p* ≤ 0.01; *** *p* ≤ 0.001.

**Table 5:** Multivariate analysis-backward elimination method based on S-25(OH)D (nmol/L) and immigration status using Cycles 3 and 4 of Canadian Health Measures Survey data.

	S-25(OH)D		
	Beta Estimate (SE)	(95% CI)	*p*-Value
Immigration status	−5.28 (1.06)	−7.48, −3.09	<0.001
Sex	4.54 (0.88)	2.71, 6.36	<0.001
Season	3.44 (0.97)	1.42, 5.46	0.002
Age	0.14 (0.04)	0.07, 0.22	0.001
Traveled to a warm/sunny climate	6.31 (1.67)	2.85, 9.78	0.001
BMI	−4.89 (0.64)	−6.11, −3.48	<0.001
Dairy products (milk, cheese, yogurt)	5.79 (0.74)	4.25, 7.32	<0.001
Skin pigmentation (melanin)	1.36 (0.28)	0.79, 1.93	<0.001
Sunscreen use	4.95 (1.12)	2.63, 7.28	<0.001
Ethnicity	−15.11 (1.61)	−18.46, −11.78	<0.001
VitD-supplement and/or analog use	0.52 (0.16)	0.17, 0.86	0.005
-Const.	29.01 (5.91)	17.91, 40.12	<0.001

Adjusted linear regression for age, sex, income, education, BMI, season, sun exposure, sunscreen use, country of birth, melanin levels, ethnicity, vitD-medication/supplements, and food consumption variables. Significant at *p* ≤ 0.05. BMI, body mass index; SE, standard error; CI, confidence interval.

## Data Availability

Data described in the manuscript, codebook, and analytic code will not be made publicly available because the data are confidential national survey data hosted by Statistics Canada.

## Appendix A

Appendix A: Supporting information regarding the steps used to combine Cycles 3 and 4, and detailed information about the calculation of the variables of interest (Table A1).

**Table A1:** Variables of interest in Cycles 3 and 4 of the Canadian Health Measures Survey.

Variable	Description
Dependent variable	We used the contentious variable serum 25(OH)D (mmol/L). We categorized vitD status using the following cut-offs for S-25(OH)D: (<30 (nmol/L); (<50 (nmol/L); (<75 (nmol/L); and (≥75 (nmol/L). First, <30 nmol/L is used to identify deficient people. Second, (<50 nmol/L) is a cut-off for the insufficient 25(OH) D, which includes the deficient and insufficient people (it is an accumulating value and not range for the insufficient people). Third, (<75 nmol/L) is a cut-off and accumulating value for deficient, insufficient, and sufficient. Fourth (≥75 (nmol/L), it is the “no added value” or the “optimal” as defined by IOM or other experts [11,12,13]. Moreover, we used ranges of S-25(OH)D for the above mentioned cut-offs (<30 nmol/L; 30–49 nmol/L; 50–74 nmol/L and ≥75 nmol/L) for sub-groups of participants.
Independent variables	
Immigration status	Landed immigrant (yes, no).
Age at time of immigration	Age at immigration was categorized as <18 years or ≥18 years.
Time since immigration	We used the continuous variable “length of time since first came to live in Canada” to categorize time since imigration at two cutoff points (≤5 years and >5 years; ≤10 years and >10 years). We used these points as indications of recent immigrants and long-term immigrants.
Sex	Male/female.
Age	Contentious and categorical variables.
Education	Education was classified as ≤secondary school or >secondary school.
Household income	Household income was derived and imputed by Statistics Canada. The contentious variable and categorical variables (<50,000, 50,000–100,000, and >100,000) were used as appropriate.
Body mass index	Calculated as weight divided by height squared (kg/m^2^). We used body mass index norms for adults (≥18 years) based on national standards for weight classification [30]. For children (3–17 years), body mass index was classified according to World Health Organization percentiles [31].
Smoking	We categorized smoking status into two groups (former/non-smoker vs. current smoker).
Alcohol	We categorized alcohol consumption into two groups (former/non-drinker vs. current drinker).
Vitamin D-containing supplements/medications	All prescription medications, over-the-counter, and herbal remedies taken in the past month (including vitD supplements and medications) were recorded in the CHMS data. The Anatomical Therapeutic Chemicals (ATCs) is a system that classifies products according to the organ and their chemical, pharmacological, and therapeutic properties. The vitD -related ATC codes were selected (A11CC01–05). These ATC codes represent ergocalciferol (vitD 2), dihydrotachysterol (synthetic vitD analog), alfacalcidol (analog of vitD), calcitriol, and cholecalciferol (vitD 3). Users of any of these were recorded as “yes,” non-users as “no,” and not respondents as “missing.”
Dietary intake	The CHMS collected dietary intake of different types of each item (red meat, liver, fish, egg, milk, cheese, yogurt, margarine) based on the previous month’s consumption. Statistics Canada prepared derived variables for each item to describe the number of times that item had been consumed/year. These derived variables were used to calculate consumption per week or day as needed. Different types of each item were merged into a single category. For example, all kinds of milk (plain, flavored, omega-3) were merged as “all milk.” The same procedure was used for “all meats” (e.g., red meat, liver, sausage, hotdog), as well as “all cheese,” “all yogurt,” “all eggs,” and “all margarine.” Another category was used for all dairy products, called “all diary.” We also used fish consumption in the past two months as (yes vs. no). Values of ≤1 time/week vs. >1 time/week were used for all categories, except all milk and all dairy, which used ≤7 times/week vs. >7 times/week.
Calcium and phosphorus	Contentious variables (mmol/L).
Pregnancy	Pregnancy was classified as Yes or No.
Ethnicity	Broadly, ethnicity was classified into two main groupings as white or non-white. Statistics Canada categorized non-white into the 12 largest ethnic groups (Aboriginal, South Asian, Chinese, Black, Filipino, Latin American, Arab, South East Asian, West Asian, Korean, Japanese, Multiple ethnicities), and another category for all other ethnicities.
Season of blood sampling	The season variable was created based on the derived variable of “month of blood sampling.” We categorized the season as winter (December–February), spring (March–May), summer (June–August), and fall (September–November).
Regions of birth	We used five regions: Canada and North America; South and Central America and the Caribbean; Europe; Africa; and Asia.
Countries of birth	We used the geographic classifications of Statistics Canada to identify the country of birth for all CHMS participants (153 countries) [58]. We ranked and selected the top 20 countries based on the number of participants (≥30 participants from each). These countries were Canada, China, the US, France, Jamaica, the UK, Algeria, Mexico, Pakistan, the Netherlands, India, the Philippines, Romania, Hong Kong, Germany, Colombia, Morocco, Italy, Iran, and Lebanon. We combined all other countries in one category, “Other,” and combined the 153 countries (excluding Canada) in another class to represent all foreign-born immigrants.
Sun exposure (10 am to 4 pm)	Sun exposure was classified as <30 min\day or ≥30 min\day.
Sunscreen application	Sunscreen application was classified as yes or no.
Clothing type	The clothing type was based on coverage and classified as yes or no. Yes indicated typically covered (face, ears, neck or arms and legs).
Physical activity	Physical activity for adults (≥18 years) was calculated based on the Canadian Physical Activity Guidelines (CPAG). The CPAG recommendation for adults is to accumulate at least 150 min of moderate-to-vigorous intensity aerobic physical activity per week, in bouts of ≥10 min. The CPAG recommendation for children (5–17 years) is to accumulate at least 60 min of moderate-to-vigorous intensity physical activity daily for at least three days per week [32].
Traveled to a sunny/warmer place (in the past two months)	Travelling to a sunny/warmer place in the past two months was classified as yes or no.
Skin pigmentation (melanin levels)	Melanin is the component that gives skin its natural color. Melanin levels were measured from the back of the hand three times; a fourth measurement was required if the difference between the first three melanin values deviated by more than 10 units. The final average calculated by Statistics Canada indicates the absolute index values of melanin, where the higher the value, the more melanin present in the epidermal. The device used for measurement was the DSM II ColorMeter (Cortex Technology, Hadsund, Denmark).

### Appendix A.1. Combining Cycles 3 and 4

The CHMS is the first national population-based survey on vitamin D (vitD) conducted in Canada. Data were collected in six cycles biannually between 2007–2019. Compared with other cycles, Cycle 3 (2012–2013) and Cycle 4 (2014–2015) are considered the most thematically consistent in terms of vitD-related sociodemographic characteristics, lifestyle factors, vitD-rich foods consumption, and behavioral, environmental, and biological determinants. Immigrant status was defined as being born outside Canada [27]. In this study, we combined data from Cycles 3 and 4 to increase the sample size and provide a more stable and precise estimate of sampling variability at the national level than was possible using a single data cycle estimate. The combination of the two cycles followed Statistics Canada’s guideline for combining CHMS cycles. As recommended, we used Bootstrap 1–Bootstrap 500, full-weight or applicable weight, and the required degrees of freedom to correct variance estimations. Therefore, the pre-specified combined full-sample weights and degrees of freedom for the two cycles were used in our analyses. The single-cycle weight was dropped from the dataset as it was a cycle-specific weight. The combined response rate (response rate for all study components, such as household visit, blood samples, and activity monitor) at the Canadian level for Cycle 3 was 51.7%, and that for Cycle 4 was 53.7% [33,34].

### Appendix A.2. Population, Comparators, Measures, and Outcome

The population of interest in this study was first-generation Canadian immigrants (foreign-born) from different origins and ethnicities. The comparators were non-immigrants (native-born population) and the white ethnic grouping. The S-25(OH)D was measured using chemiluminescence immunoassay technology (DiaSorin^®^, Ltd., Stillwater, Minnesota, USA). The analytical detection limit for S-25(OH)D was 10–375 nmol/L. The inter-assay coefficients of variation for the S-25(OH)D was 13.0% and the precision for <20 nmol/L, 20–100 nmol/L and >100 nmol/L levels were 15.0%, 10.0% and 12.0%, respectively [45].

The results are presented as weighted means or proportions only. The weighted results and proportions were based on the total number of 11,579 participants. The number of participants for each group cannot be published because of Statistics Canada’s restrictions policy relates to publishing weighted results. The weighting technique for complex survey results refers to statistical adjustments that have been used to correct and improve the accuracy of the survey estimates. The survey weights adjust for unequal probabilities of selection that often have occurred during sampling design and to help compensate for survey non-response weights. Thus, by using the survey weights to create estimates should yield approximately unbiased national prevalence estimates and reduce non-response bias that can be introduced due to random missing values. The study outcomes were the serum 25(OH)D levels and vitD status.

### Appendix A.3. Variables of Interest

The data manipulation and all derived variables were consistent with Statistics Canada’s instructions based on the specific cycle user guide, data codebook, instructions for combining more than one cycle, and the derived variable specifications [33]. The CHMS microdata are hosted in secure centers overall in Canada. These data were accessed and analyzed at the Carleton, Ottawa, Outaouais Local Research Data Centre (COOLRDC), located in the Morisset Library at the University of Ottawa.

## Appendix B

Appendix B: supporting information including (Table A2, Table A3, Table A4, Table A5 and Table A6).

This Appendix sets out the steps followed in combining Cycles 3 and 4 of the Canadian Health Measures Survey (CHMS), variables of interest, critical data manipulation stages, and other methodological aspects.

This Appendix contains supplementary material for the manuscript entitled “*Vitamin D Status among First-Generation Immigrants from Different Ethnic Groups and Origins: An Observational Study using the Canadian Health Measures Survey.*”

**Table A2:** Weighted prevalence and mean S-25(OH)D (nmol/L) levels and basic study characteristics using Cycles 3 and 4 of Canadian Health Measures Survey data.

		Cycle 3 (49.52%) %	Cycle 4 (50.48%) %	Total (100%) %
Immigration status	Immigrant	24.5	19.38	21.90
Ethnic group	White	75.10	78.87	76.98
Sex	Female	50.00	50.10	50.08
Age (years)	<5	2.47	2.14	2.30
	5–11	7.92	8.17	8.04
	12–17	7.58	7.21	7.39
	18–64	70.70	70.29	70.54
	>64	11.20	12.20	11.72
Household income (CAD)	<50,000	38.10	32.91	35.45
	50,000–100,000	36.00	37.26	36.61
	>100,000	26.00	29.84	27.93
S-25(OH)D status (nmol/l)	<50	65.04	62.27	63.64
Weighted Mean		Mean (SE)	Mean (SE)	Mean (SE)
	Age (years)	39.08 (0.15)	39.35 (0.09)	39.23 (0.085)
	S-25(OH)D (nmol/L)	61.29 (2.78)	59.16 (1.99)	60.28 (1.69)

SE, standard error.

**Table A3:** Weighted prevalence of vitamin D-rich food/fish consumption for immigrants and non-immigrants using Cycles 3 and 4 of Canadian Health Measures Survey data.

		Non-Immigrants (78.9%), %	Immigrants (21.9%), %	All Participants (100%), %	*p*-Value
All meats (red meat, liver, hotdog, and sausage)	>1 time/week	90.47	78.72	87.89	<0.001
All eggs (yolk/omega-3)	>1 time/week	59.68	66.95	61.26	0.067
All milk (plain, flavored, and omega-3)	>7 times/week	54.63	48.38	53.27	0.027
All cheese (cottage and other)	>1 time/week	86.70	63.26	81.57	<0.001
All yogurt	>1 time/week	72.19	70.15	71.75	0.577
All dairy products (milk, yogurt, and cheese)	>7 times/week	83.38	75.38	81.64	<0.001
All margarine (plain and omega-3)	>1 time/week	84.11	70.95	81.90	0.007
Fortified juice with calcium/vitamin D	>1 time/week	7.01	6.12	6.81	0.452
Fish consumption/past 2 months	Yes (No ^†^)	25.18	17.09	23.43	0.046

Reference values: ≤1 time/week; ≤7 times/week. ^†^ Reference value; *p*-values ≤ 0.05.

**Table A4:** Weighted means for S-25(OH)D (nmol/L) and vitamin D status by sociodemographic and lifestyle factors using Cycles 3 and 4 of Canadian Health Measures Survey data.

		S-25(OH)D (nmol/L)			S-25(OH)D Status			
		Mean (SE)	(95% CI)	*p*-Value	<30 (nmol/L (10.3%),%	<50 (nmol/L (63.64%),%	<75 (nmol/L (76.1%),%	≥75 (nmol/L (23.9%),%
Household income (CAD)	<50,000 ^†^	56.18 (1.90)	-		13.95	45.10	79.43	20.57
	50,000–100,000	61.44 (1.81)	(−7.73, −2.79)	<0.001	9.67	33.14	76.02	23.98
	>100,000	63.66 (1.79)	(−9.97, −4.98)	<0.001	6.53 ***	29.64 ***	72.09	27.91 **
Education	≤Secondary school ^†^	59.41 (1.64)			9.57	36.81	77.58	22.42
	>Secondary school	61.00 (1.86)	(−3.59, 0.41)	0.113	10.87	35.83	74.97	25.03
Pregnancy	No^†^	60.35 (2.15)	-		11.72	36.88	75.29	24.71
	Yes	51.22 (7.40)	(−6.69, 24.94)	0.244	2.11	45.70	93.21	6.79 *
Smoking habits	Former/non-smoker ^†^	61.59 (1.66)	-		9.98	34.12	73.82	26.18
	Current smoker	53.09 (2.12)	(5.74, 11.24)	<0.001	14.88 *	50.71 ***	85.02	14.98 ***
Alcoholic beverages	Former/non-drinker ^†^	54.25 (2.17)	-		15.72	38.15	81.44	18.56
	Current drinker	61.33 (1.67)	(−10.49, 3.66)	<0.001	9.73 **	34.70 ***	74.70	25.30 **
Meet the physical activity recommendations	No ^†^	58.79 (1.54)	-		11.32	39.34	77.91	22.09
	Yes	63.80 (2.34)	(−8.26, 1.76)	0.004	8.01	29.54 ***	72.86	27.14 *
Clothing	Uncovered ^†^	62.07 (1.69)	-		8.23	31.13	74.85	25.15
	Typically covered	57.91 (2.00)	(1.46, 6.85)	0.004	12.86 **	42.58 ***	77.86	22.14

^†^ Reference value; SE, standard error; CI, confidence interval; BMI, body mass index. *p*-values: * *p* ≤ 0.05; ** *p* ≤ 0.01; *** *p* ≤ 0.001.

**Table A5:** Weighted means for S-25(OH)D (nmol/L) by season and month of blood test using Cycles 3 and 4 of Canadian Health Measures Survey data.

		S-25(OH)D (nmol/L)		*p*-Value
		Mean (SE)	(95% CI)	
Season	Winter **^†^**	53.80 (2.47)	-	
	Spring	54.63 (1.41)	(−7.16, 5.49)	0.786
	Summer	66.13 (2.38)	(−18.50, −6.16)	<0.001
	Fall	66.00 (3.63)	(−20.59, −3.83)	0.006
Month	January **^†^**	57.67 (9.25)	-	
	February	47.13 (6.17)	(−25.73, 4.65)	0.164
	March	52.02 (9.57)	(−28.63, 18.86)	0.674
	April	57.31 (1.64)	(−23.46, 3.10)	0.126
	May	55.00 (2.17)	(−21.38, 5.63)	0.240
	June	63.34 (8.61)	(−38.14, 5.73)	0.140
	July	68.02 (5.41)	(−37.88, −3.89)	0.018
	August	67.78 (7.31)	(−40.77, −0.53)	0.045
	September	72.08 (10.24)	(−49.86, −0.04)	0.050
	October	67.34 (5.39)	(−37.99, −2.42)	0.028
	November	57.52 (2.80)	(−24.80, −4.02)	0.149
	December	59.20 (13.18)	(−42.89, 18.75)	0.425

^†^ Reference value; SE, standard error; CI, confidence interval; *p*-values ≤ 0.05.

**Table A6:** Weighted mean S-25(OH)D concentrations (nmol/L) by immigration status and season using Cycles 3 and 4 of Canadian Health Measures Survey data.

		Non-Immigrant ^†^	Immigrant		
		Mean (SE)	Mean (SE)	(95% CI)	*p*-Value
Season	Winter	55.36 (2.33)	47.79 (4.55)	(−0.67, 15.82)	0.070
	Spring	56.94 (1.62)	47.71 (3.07)	(2.27, 16.19)	0.012
	Summer	70.03 (2.23)	55.21 (1.76)	(9.28, 20.36)	<0.001
	Fall	68.11 (3.55)	54.87 (3.95)	(8.00, 18.47)	<0.001

^†^ Reference value; SE, standard error; CI, confidence interval; *p*-values ≤ 0.05.

## References

[1] Charoenngam N., Shirvani A., Holick M.F. (2019). Vitamin D for skeletal and non-skeletal health: What we should know. J. Clin. Orthop. Trauma.

[2] Shin S.Y., Kwon M.J., Song J., Park H., Woo H.Y. (2013). Measurement of Serum Total Vitamin D (25-OH) Using Automated Immunoassay in Comparision With Liquid Chromatography Tandem-Mass Spectrometry. J. Clin. Lab. Anal..

[3] Atef S.H. (2018). Vitamin D assays in clinical laboratory: Past, present and future challenges. J. Steroid Biochem. Mol. Biol..

[4] (2018). Martens Immunoassay for Free Vitamin D. https://patentimages.storage.googleapis.com/57/7e/64/9c4548d4ed8ce1/US9897615.pdf.

[5] Nowson C.A., McGrath J.J., Ebeling P.R., Haikerwal A., Daly R.M., Sanders K.M., Seibel M.J., Mason R.S. (2012). Vitamin D and health in adults in Australia and New Zealand: A position statement. Med. J. Aust..

[6] Heaney R.P., Recker R.R., Grote J., Horst R.L., Armas L.A. (2011). Vitamin D3 is more potent than vitamin D2 in humans. J. Clin. Endocrinol. Metab..

[7] De Boer I.H., Kestenbaum B., Shoben A.B., Michos E.D., Sarnak M.J., Siscovick D.S. (2009). 25-hydroxyvitamin D levels inversely associate with risk for developing coronary artery calcification. J. Am. Soc. Nephrol..

[8] Spiro A., Buttriss J. (2014). Vitamin D: An overview of vitamin D status and intake in E urope. Nutr. Bull..

[9] Zmijewski M.A. (2019). Vitamin D and Human Health. Int. J. Mol. Sci..

[10] Mithal A., Wahl D.A., Bonjour J.-P., Burckhardt P., Dawson-Hughes B., Eisman J.A., Fuleihan G.E.-H., Josse R.G., Lips P., Morales-Torres J. (2009). Global vitamin D status and determinants of hypovitaminosis D. Osteoporos. Int..

[11] Ross A., Taylor C., Yaktine A., Del Valle H. (2011). Dietary Reference Intakes: Calcium and Vitamin D. Committee to Review Dietary Reference Intakes for Vitamin D and Calcium Food and Nutrition Board.

[12] Vieth R. (2011). Why the minimum desirable serum 25-hydroxyvitamin D level should be 75 nmol/L (30 ng/mL). Best Pract. Res. Clin. Endocrinol. Metab..

[13] Dawson-Hughes B. (2014). Vitamin D Deficiency in Adults: Definition, Clinical Manifestations, and Treatment.

[14] Pham T. (2012). Vitamin D Status of Immigrant and Ethnic Minority Children Ages 2 to 5 y in Montréal. Master’s Thesis.

[15] Janz T., Pearson C. (2013). Vitamin D Blood Levels of Canadians.

[16] Yousef S., Elliott J., Manuel D., Colman I., Papadimitropoulos M., Hossain A., Leclair N., Wells G.A. (2019). Study protocol: Worldwide comparison of vitamin D status of immigrants from different ethnic origins and native-born populations—A systematic review and meta-analysis. Syst. Rev..

[17] Cashman K.D., Dowling K.G., Škrabáková Z., Gonzalez-Gross M., Valtueña J., De Henauw S., Moreno L., Damsgaard C.T., Michaelsen K.F., Mølgaard C. (2016). Vitamin D deficiency in Europe: Pandemic?. Am. J. Clin. Nutr..

[18] Martin C.A., Gowda U., Renzaho A.M. (2016). The prevalence of vitamin D deficiency among dark-skinned populations according to their stage of migration and region of birth: A meta-analysis. Nutrition.

[19] Eggemoen Å.R., Knutsen K.V., Dalen I., Jenum A.K. (2013). Vitamin D status in recently arrived immigrants from Africa and Asia: A cross-sectional study from Norway of children, adolescents and adults. BMJ Open.

[20] Chui T., Statistics Canada (2011). Immigration and Ethnocultural Diversity in Canada: National Household Survey.

[21] Salant T., Lauderdale D.S. (2003). Measuring culture: A critical review of acculturation and health in Asian immigrant populations. Soc. Sci. Med..

[22] Zhu L. (2017). Depression risks and correlates among different generations of Chinese Americans: The effects of relationships with friends and relatives. Soc. Sci..

[23] Stephens W., Klimiuk P., Warrington S., Taylor J., Berry J., Mawer E. (1982). Observations on the natural history of vitamin D deficiency amongst Asian immigrants. QJM Int. J. Med..

[24] Stern P.J. (2002). Generational differences. J. Hand Surg..

[25] Von Elm E., Altman D.G., Egger M., Pocock S.J., Gøtzsche P.C., Vandenbroucke J.P. (2007). The Strengthening the Reporting of Observational Studies in Epidemiology (STROBE) statement: Guidelines for reporting observational studies. Bull. World Health Organ..

[26] Day B., Langlois R., Tremblay M., Knoppers B.-M. (2007). Canadian Health Measures Survey: Ethical, legal and social issues. Health Rep..

[27] Ng E. (2015). Canadian Health Measures Survey: A tool for immigrant health research?. Health Rep..

[28] Statistics Canada Canadian Health Measures Survey, Cycle 4, 2014–2015—Privacy Impact Assessment Summary. https://www.statcan.gc.ca/eng/about/pia/chmsc4.

[29] Statistics Canada 2012–2013 Canadian Health Measures Survey, Cycle 3—Privacy Impact Assessment. https://www.statcan.gc.ca/eng/about/pia/chmsc3.

[30] Health Canada (2003). Canadian Guidelines for Body Weight Classification in Adults—Quick Reference Tool for Professionals.

[31] Onis M.d., Onyango A.W., Borghi E., Siyam A., Nishida C., Siekmann J. (2007). Development of a WHO growth reference for school-aged children and adolescents. Bull. World Health Organ..

[32] Canadian Physical Activity (2012). Sedentary Behaviour Guidelines.

[33] Statistics Canada (2015). Canadian Health Measures Survey (CHMS)Data User Guide: Cycle 3. https://usermanual.wiki/Pdf/CHMSUserGuideCycle3E.484319040/help.

[34] Statistics Canada Canadian Health Measures Survey (CHMS)—Cycle 4. https://www23.statcan.gc.ca/imdb/p2SV.pl?Function=getSurvey&Id=148760.

[35] Hilger J., Friedel A., Herr R., Rausch T., Roos F., Wahl D.A., Pierroz D.D., Weber P., Hoffmann K. (2014). A systematic review of vitamin D status in populations worldwide. Br. J. Nutr..

[36] Pludowski P., Grant W.B., Bhattoa H.P., Bayer M., Povoroznyuk V., Rudenka E., Ramanau H., Varbiro S., Rudenka A., Karczmarewicz E. (2014). Vitamin D status in central Europe. Int. J. Endocrinol..

[37] Lips P. (2007). Vitamin D status and nutrition in Europe and Asia. J. Steroid Biochem. Mol. Biol..

[38] Alshahrani A.A. (2014). Vitamin D Deficiency and Possible Risk Factors among Middle Eastern University Students in London, Ontario, Canada. Master’s Thesis.

[39] McCormack D., Mai X., Chen Y. (2017). Determinants of vitamin D supplement use in Canadians. Public Health Nutr..

[40] Knoss R., Halsey L.G., Reeves S. (2012). Ethnic dress, vitamin D intake, and calcaneal bone health in young women in the United Kingdom. J. Clin. Densitom..

[41] Ojah R.C., Welch J.M. (2012). Vitamin D and musculoskeletal status in Nova Scotian women who wear concealing clothing. Nutrients.

[42] Mark S. (2010). Vitamin D Status and Recommendations to Improve Vitamin D Status in Canadian Youth. Master’s Thesis.

[43] Naugler C., Zhang J., Henne D., Woods P., Hemmelgarn B.R. (2013). Association of vitamin D status with socio-demographic factors in Calgary, Alberta: An ecological study using Census Canada data. BMC Public Health.

[44] Ghasemian R., Shamshirian A., Heydari K., Malekan M., Alizadeh-Navaei R., Ebrahimzadeh M.A., Jafarpour H., Shahmirzadi A.R., Khodabandeh M., Seyfari B. (2020). The Role of Vitamin D in The Age of COVID-19: A Systematic Review and Meta-Analysis. MedRxiv.

[45] Munasinghe L.L., Yuan Y., Willows N.D., Faught E.L., Ekwaru J.P., Veugelers P.J. (2017). Vitamin D deficiency and sufficiency among Canadian children residing at high latitude following the revision of the RDA of vitamin D intake in 2010. Br. J. Nutr..

[46] Wang L., Manson J.E., Buring J.E., Lee I.-M., Sesso H.D. (2008). Dietary intake of dairy products, calcium, and vitamin D and the risk of hypertension in middle-aged and older women. Hypertension.

[47] Thuesen B., Husemoen L., Fenger M., Jakobsen J., Schwarz P., Toft U., Ovesen L., Jørgensen T., Linneberg A. (2012). Determinants of vitamin D status in a general population of Danish adults. Bone.

[48] Renzaho A.M., Halliday J.A., Nowson C. (2011). Vitamin D, obesity, and obesity-related chronic disease among ethnic minorities: A systematic review. Nutrition.

[49] Neale R., Khan S., Lucas R., Waterhouse M., Whiteman D., Olsen C. (2019). The effect of sunscreen on vitamin D: A review. Br. J. Dermatol..

[50] Young A., Narbutt J., Harrison G., Lawrence K., Bell M., O’Connor C., Olsen P., Grys K., Baczynska K., Rogowski-Tylman M. (2019). Optimal sunscreen use, during a sun holiday with a very high ultraviolet index, allows vitamin D synthesis without sunburn. Br. J. Dermatol..

[51] Brooks S.P., Greene-Finestone L., Whiting S., Fioletov V.E., Laffey P., Petronella N. (2017). An analysis of factors associated with 25-hydroxyvitamin D levels in white and non-white Canadians. J. AOAC Int..

[52] Nimitphong H., Holick M.F. (2013). Vitamin D status and sun exposure in Southeast Asia. Derm. Endocrinol..

[53] Sanou D., O’Reilly E., Ngnie-Teta I., Batal M., Mondain N., Andrew C., Newbold B.K., Bourgeault I.L. (2014). Acculturation and nutritional health of immigrants in Canada: A scoping review. J. Immigr. Minority Health.

[54] Carlberg C. (2019). Nutrigenomics of vitamin D. Nutrients.

[55] Leandro A., Rocha M., Cardoso C., Bonecini-Almeida M. (2009). Genetic polymorphisms in vitamin D receptor, vitamin D-binding protein, Toll-like receptor 2, nitric oxide synthase 2, and interferon-γ genes and its association with susceptibility to tuberculosis. Braz. J. Med Biol. Res..

[56] Aucoin M., Weaver R., Thomas R., Jones L. (2013). Vitamin D status of refugees arriving in Canada: Findings from the Calgary Refugee Health Program. Can. Fam. Physician.

[57] Baauw A., Kist-van Holthe J., Slattery B., Heymans M., Chinapaw M., van Goudoever H. (2019). Health needs of refugee children identified on arrival in reception countries: A systematic review and meta-analysis. BMJ Paediatr. Open.

[58] Statistics C. Standard Classification of Countries and Areas of Interest (SCCAI). https://publications.gc.ca/site/eng/9.856211/publication.html.

